# Efficacy of “High in” Nutrient Specific Front of Package Labels—A Retail Experiment with Canadians of Varying Health Literacy Levels

**DOI:** 10.3390/nu12103199

**Published:** 2020-10-20

**Authors:** Elizabeth D. Mansfield, Dominique Ibanez, Fuqi Chen, Emily Chen, Elaine de Grandpré

**Affiliations:** 1 Bureau of Nutritional Sciences, Food Directorate, Health Canada, Ottawa, ON K1A 0K9, Canada; emily.chen@canada.ca (E.C.); elaine.degrandpre@canada.ca (E.d.G.); 2 Bureau of Food Surveillance and Research Integration, Food Directorate, Health Canada, Ottawa, ON K1A 0K9, Canada; dominique.ibanez@canada.ca (D.I.); fuqi.chen@canada.ca (F.C.)

**Keywords:** front-of-package labels, health literacy, food choices

## Abstract

Background: In 2018, Health Canada, the Federal department responsible for public health, put forward a regulatory proposal to introduce regulations requiring a “High in” front-of-package label (FOPL) on foods that exceed predetermined thresholds for sodium, sugars, or saturated fat. This study evaluated the efficacy of the proposed FOPL as a quick and easy tool for making food choices that support reduction in the intakes of these nutrients. Methods: Consumers (*n* = 625) of varying health literacy levels (HL) were assigned to control (current labeling with no FOPL) or one of four FOPL designs. They completed six shopping tasks, designed to control for internal motivations. Efficacy was measured with correct product selection and response time (seconds) to make food choices using repeated measures statistical modeling, adjusting for HL, task type, and task order. Eye-tracking and structured interviews were used to gather additional insights about participants’ choices. Results: Overall, FOPL was significantly more effective than current labeling at helping consumers of varying HL levels to identify foods high in nutrients of concern and make healthier food choices. All FOPL were equally effective. Conclusions: “High in” FOPL can be effective at helping Canadians of varying HL levels make more informed food choices in relation to sugars, sodium, and saturated fat.

## 1. Introduction

Chronic disease is a major public health concern [1] and unhealthy diets with high levels of sodium, sugars, saturated and trans fats are one of the top risk factors for this chronic disease burden in Canada [2]. While federally regulated nutrition labeling on foods [3] is a key source of nutrition information to make informed food choices [4], many Canadians face challenges in accessing, understanding, and evaluating food label information [5,6] and more specifically identifying if a food is high in nutrients of public health concern [7]. It has been suggested that further labelling measures are needed to help protect Canadians from the risks of chronic diseases related to excess consumption of foods high in nutrients of public health concern [5,8], in particular those that may be disadvantaged by risks of limited health literacy [9].

In 2016, the World Health Organization (WHO) recommended implementing interpretive front-of-package labeling (FOPL) systems using symbols and nutrient criteria to indicate that a product has certain nutritional characteristics [10]. These FOPL systems offer consumers a simplified and visible indicator to help consumers make informed food choices, particularly among those who are limited by time, nutrition knowledge and health literacy (HL) [9,11,12]. Improving the accessibility of nutrition information and people’s capacity to understand and use that information in the context of their dietary goals and needs promotes individual health and nutrition literacy at functional, interactive and critical levels [13]. Any effort to increase the effectiveness of nutrition information systems supporting healthy eating practices and decreasing disparities in population health may be limited if these health literacy skills are not addressed [5,14,15].

While the vast majority of FOPL systems present an interpretive (e.g., France’s Nutri-score [16], Australia’s Health Star Rating [17]) or nutrient-specific (e.g., Food Industry’s Guideline daily amounts [18], the UK’s color-coded GDA [19]) nutrient profile of the relative healthiness of foods [20,21], a new paradigm for FOPL, known as nutritional warnings, have been designed to help people limit their intake of foods that contain high amounts of critical nutrients of public health concern (i.e., sodium, sugars, and saturated fat). Recent research has shown that a “High in” nutrient based FOPL is an effective consumer tool for providing more accessible and comprehensible nutrition information to consumers [22], helping them to limit nutrients of concern [23] and also enhancing perceptions of control over the healthiness of their food decisions [24]. In 2016, Chile became the first country to implement mandatory “high in” FOPL designed to help people limit their intake of foods that contain high amounts of critical nutrients of public health concern (i.e., sodium, sugars, and saturated fat) [25,26]. Other countries have recently developed or implemented similar approaches including Israel [27], Brazil [28], Peru [29], and Uruguay [30,31].

In 2018, as a key initiative under Canada’s Healthy Eating Strategy (HES) [32], Health Canada put forward a regulatory proposal to introduce regulations requiring a “High in” FOPL on foods that exceed predetermined thresholds for sodium, sugars or saturated fat. This was followed by a national consultation on four “High in” FOPL designs differing in the use of color, text, and nature of the icons [33]. The main objective of this present work was to determine if “High in” nutrient-specific FOPL approaches, as compared to current labeling, could improve consumer capacity to identify foods high in nutrients of public health concern and differentiate between healthful and less healthful products to make informed choices to reduce intakes of foods high in sodium, sugars, and/or saturated fat. Of particular interest was the efficacy among those disadvantaged by risks of marginal health literacy, who face challenges in accessing, understanding, and evaluating nutrition labelling information when making food choices [9,34] A secondary objective was to determine if a particular FOPL design element (i.e., the use of color versus black and white elements, the use of text versus icons, or the nature of an icon integrated into the FOPL) more effectively captured the attention of and communicated to consumers of varying HL levels about foods high in nutrients of public health concern [35]. This research will contribute a Canadian perspective on the efficacy of a “High in” front-of-pack labelling tool to help consumers easily and correctly identify foods high in sodium, sugars and/or saturated fat, differentiate between healthful and less healthful products, and make healthier food choices. Findings will inform Health Canada’s regulatory FOPL proposal for labelling of foods “high in” nutrients of public health concern.

## 2. Materials and Methods

A Canadian-based research organization (CRO) Explorer Research (https://explorerresearch.com/), specializing in the design and implementation of retail food lab market research experiences and eye-tracking technology, was contracted to implement the study design.

### 2.1. Study Sample and Recruitment

Participants aged 16 years and older who were responsible for their household grocery shopping (sole or shared), who had no links with food industry or Health Canada, who did not work in food and nutrition, and who had not worked previously in market research were eligible to participate. The CRO recruited participants from the Greater Toronto Area (GTA) using their market research panels. At least 10% of the participants in the current labeling and each FOPL arm were Francophone. A conscious effort was made to include Indigenous people and vulnerable groups, such as seniors and youth. The Newest Vital Sign tool, a HL tool adapted for use by Health Canada [28] was used to screen for HL during recruitment to ensure that the pool of respondents reflected the HL status of Canadians (approximately 60% marginal vs. 40% adequate) [36].

### 2.2. Research Ethics and Consent

The study was conducted according to the Tri-Council Policy Statement (TCPS), Ethical Conduct for Research Involving Humans, which sets the standard for research ethics boards in Canada. The protocol of this study was approved by Health Canada’s Research Ethics Board for Human Research (REB 2017-0033). All participants provided written informed consent before randomization into the study and following completion received financial compensation up to $50 in accordance with the normal CRO incentive.

### 2.3. Study Design

**Figure 1:**
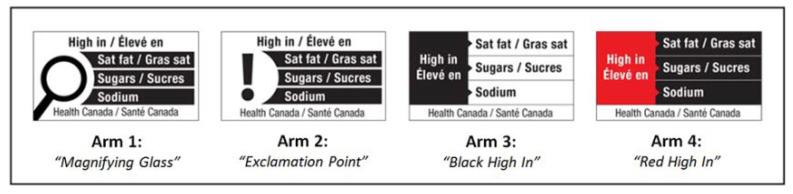
Four front-of-package label (FOPL) design arms.

This five-arm (control: current regulated nutrition labeling with no FOPL; four FOPL design arms added to current regulated nutrition labeling; Figure 1) retail experiment made use of a retail food lab to emulate a food shopping experience. The four FOPL arms were designs that had been put forward by Health Canada in their national consultation on FOPL [37]

### 2.4. Study Setting

**Figure 2:**
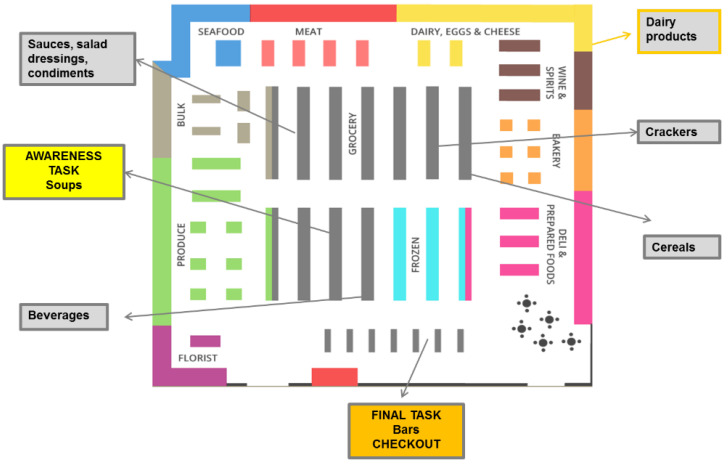
Retail food lab set-up.

The entire study was conducted between February and March 2018 in the CRO’s retail food lab located in the Greater Toronto area (Ontario, Canada). The retail food lab was customized to replicate a retail food shopping experience, including typical retail grocery shelving containing a variety of food product categories, grocery carts, and a check-out lane (Figure 2).

Food categories selected for this research were commonly consumed in Canada and represented different food uses, specifically convenience (soup), part of a meal (cereal), sweet or savory snacks (granola bar, crackers), sweet beverages (fruit drinks and juices), condiments (salad dressing), and dairy foods (yogurt) [38,39]. Food packages reflected the labeling approach of the experimental arm to which participants were assigned (current labeling or one of four FOPL arms). The remaining shelves of the lab were populated with ancillary products to ensure that the retail food lab looked and functioned as a supermarket except that participants did not pay for their food choices and no food products were taken home by participants.

Health Canada provided the contract research organization with a list of food products to include for each of the seven food categories in the research. Each product listed included detailed regulatory specifications on the type of FOPL that should be used including size level of the FOPL, dimensions of the FOPL, and the “High In” nutrients within the FOPL. Additionally, on a product-by-product basis, changes were applied to the existing package design to align with FOPL guidelines to ensure that existing health or nutrient content claims on the pack did not supersede the claims made in the FOPL (Figure S1). These changes were accomplished through relocation on the front of package and/or reduction in size. The FOPL and additional customizations were applied across all applicable packages in the category and the new label replaced or covered the existing front panel of the package. Where bilingual packaging had 2 sides (French and English), two labels were produced in the necessary language and affixed to the product. In cases where a continuous label was present, e.g., soup, a continuous new label was produced and affixed to the existing product.

### 2.5. Study Procedures

After obtaining consent, participants were randomly assigned using the CRO’s computer-generated random allocation sequencing program to either current labeling (control) or a FOPL design arm. The randomization program was coded to generate the 60:40 marginal/adequate HL split and to balance the arms for sociodemographic characteristics of gender, age and language (French/English). Participants were exposed only to the labeling intervention that they were assigned to and were blinded to all other interventions. For example, those participants in the control group saw food packages with the current regulated nutrition labeling (i.e., Nutrition Facts table (NFt), list of ingredients (LOI)) and those in the FOPL arm 1 were exposed to the current regulated nutrition labelling with the addition of the FOPL with the magnifying glass (Figure 1) on any food packages that were high in sodium, sugars, and/or saturated fat.

Participants completed six shopping tasks consisting of two non-specific tasks, two specific tasks and two findability tasks (Table 1). For all shopping tasks participants wore eye-tracking glasses to objectively assess what nutrition label information they noticed, as well as how much time they spent on different components of food label information when making each food task choice. The nature of the different shopping tasks allowed study researchers to better account for motivating factors of food choices, more specifically: non-specific shopping tasks to contextualize for consumer’s intrinsic motivation for food choices (e.g., choose a yogurt for your household); specific shopping tasks to contextualize for shopping with a pre-defined dietary need (e.g., choose a cereal for someone trying to cut down on their sugar intake); and findability shopping tasks to determine how FOPL help consumers identify foods high in nutrients of public health concern and distinguish between foods when making food choices (e.g., find any cracker high in sodium and saturated fat). An introductory task ensured that those participants in the FOPL arms were exposed to different variants of their assigned FOPL arm, including soups that were high in one, two, or all three nutrients of public health concern.

A trained interviewer led each participant through the shopping experience using a hand-held computer tablet with a pre-scripted shopping interview guide. The interview guide was programmed with a randomization sequence by shopping task type and food category. This randomization ensured that each participant shopped each food category and completed each of the different task types with a different food category. With the exception of the introductory task which was always conducted with the soup category, and the final findability task at the check-out counter which was always conducted with the granola bar category, each food category was randomly assigned without replacement for subsequent shopping tasks (Table 2). This means that once a food category was shopped by a participant it could not be shopped again for any other task.

Each shopping task was read out loud by the interviewer at point of arrival at the shelf representing the food category to be shopped. At this point, the time to complete each shopping task was recorded up until the consumer placed their food choice into the shopping basket. On completion of each shopping task, interviewers used a structured interview guide to gain insights on consumer awareness, understanding, appraisal, and use of information on food labels to make each of their food choices. The duration of the shopping experience and interview took approximately an hour to complete with each participant.

### 2.6. Outcomes

The primary objective was to determine if “High in” nutrient specific FOPL, as compared to current labeling, could improve consumer capacity to identify foods high in nutrients of public health concern (i.e., saturated fat, sugars, sodium) and differentiate between healthful and less healthful products to make informed choices to reduce intakes of foods high in sodium, sugars, and/or saturated fat. Successful decision-making for each shopping task included choosing foods that are not high in saturated fat, sodium, and sugars (non-specific task), choosing foods to decrease intakes of saturated fat, sugars, and/or sodium (specific tasks), and finding foods high in saturated fat, sugars, and/or sodium (findability tasks). Efficacy was measured with response time (seconds) to make a successful food choice, i.e., to choose foods with saturated fat, sugars, and sodium levels below predetermined thresholds for the specific and non-specific tasks or to identify foods high in saturated fat, sugars, and/or sodium for the findability tasks.

A secondary objective 2 was to determine which particular FOPL design elements (i.e., the use of color versus black and white elements, the use of text versus icons, or the nature of an icon integrated into the FOPL) more effectively captured the attention of and communicated to consumers of varying HL levels about foods high in nutrients of public health concern. This was evaluated with consumer attentional capture of package features; visual processing using time to first fixation (TTTFF) on areas of interest (AOI) on the food label (i.e., FOPL and Nutrition Facts table (NFt)); fixation duration; and number of fixations. Data on awareness and understanding of each FOPL collected through responses to interview questions.

### 2.7. Sample Size

A pilot study of the full research protocol was conducted with 130 consumers (current labeling *n* = 62; one FOPL arm *n* = 68). The observed success rates in the control and FOPLs arms were 73% and 79%, respectively. The observed times to make a selection were 50 ± 39 s in the control group and 40 ± 39 s in the FOPL arm. Using these results, the sample size needed for the entire study to determine a detectable difference in success outcomes of 20% at a 95% significance level and an 80% power was determined to be *n* = 625 (*n* = 125/arm).

### 2.8. Data Analysis

#### 2.8.1. Shopping Experiment Data Analysis

Results were summarized by study group, task type (introductory soup task excluded), and HL status. Pooling of the results (i.e., food choices and shopping time to make food choices) by task type from the four FOPL arms was conducted to determine if FOPL provided quick and easy guidance compared to current labeling (500 participants in FOPL arms compared to 125 participants in current labeling) for each task type across all tasks, and by HL status. Food choices and shopping time to make each food choice were also compared between the four FOPL arms (groups of 125 participants compared to each other) for each task type and overall.

Logistic regression modeling (with repeated measures for tasks 2 and 3) was undertaken to investigate successful food choices (excluding the introductory task) taking into consideration the labeling conditions, the different task types, task order and consumers’ HL levels. For each task, two logistic regression models on success were run, one for comparison between control and FOPL arms combined, and another for the FOPL arms (using arm1 as baseline). Overall comparisons of success combining all 3 tasks together were then conducted using logistic regression models for comparison between control and FOPL arms combined, and another for the FOPL arms (using arm1 as baseline).

Additional mixed effect linear regression models (with repeated measures for tasks 2 and 3) were conducted for shopping time to make successful food choices (excluding the introductory task) taking into consideration the labeling conditions, the different task types, task order and consumers’ HL levels. For each task, two regression models on shopping time for successful food choices were run, one for comparison between control and FOPL arms combined, and another for the FOPL arms (using arm1 as baseline). Overall comparisons of shopping time to make successful food choices combining all 3 tasks together were then conducted using regression models for comparison between control and FOPL arms combined, and another for the FOPL arms (using arm1 as baseline). Distributions of shopping times were not normally distributed, so the modeling procedure was also performed on Box-Cox transformed data and Wilcoxon nonparametric measures were used to confirm the significance of the treatment effect (Control vs. FOPL combined and for each FOPL arm).

#### 2.8.2. Eye Tracking Data Analysis

Results for objective 2, attentional capture of FOPL, were examined by evaluating the time spent on AOI for all participants and participants with positive fixations on AOI, by FOPL arm, task type (introductory task excluded), overall, and by success and failure. Eye-tracking data for TTTFF on AOI on the front and back of food packages were compared between the FOPL arms for each task type. This analysis was repeated for those who made successful food selections. Eye-tracking data was analyzed to show if and for how long participants looked at the FOPL when making their food choices. Interview responses were compared to determine efficacy of specific FOPL design elements for quick and easy guidance and food decision-making.

Eye-tracking data were skewed and required square root transformation to increase data normality. Box-Cox transformation method was applied to the shopping time to increase normality. A regression model was fitted with the transformed time set to be the response variable. Covariates of interest included study group (main effect), HL (candidate variable), and the order of trials (for specific and findability tasks). For non-specific tasks we used the classical regression model as there was only one trial. There were repeated trials for specific and findability tasks, so mixed effect modeling with repeated measures (on the order of trials) together with unstructured covariance matrix was used. The *p*-values and significance status of the effects were examined based on the type 3 analysis of effects. Final model selection was based on the *p*-values of the effects, thus only the main effect and the significant candidate effects were kept in the final model. Based on the final model, we also used Least Squared means pairwise comparisons with Bonferroni’s adjustment to study the difference between the FOPL arms. Box-Cox transformations for participants with positive fixations were used to analyze the total time spent on FOPL and NFt. The modeling analysis was conducted for each of the three tasks overall and for success only. The results were confirmed by Wilcoxon rank-sum tests.

#### 2.8.3. Interview Data Analysis

Summary statistics for participants’ self-reported use of FOPL, NFt, LOI, and health claims for overall food choices and for successful choices only were derived from the specific interview questions for each task type. Logistic regression modeling (with repeated measures for tasks 2 and 3) was used to analyze the impact of self-reported use of FOPL on success rates for each task type. Summary statistics, logistic regression modeling (with repeated measures for tasks 2 and 3), and contrast pairwise testing were used to analyze the relationships between the self-reported usage of brand name/product image and the usage of FOPL when making successful shopping decisions for each task type. Logistic regression modeling (with repeated measures for tasks 2 and 3) was repeated to compare pairs of FOPLs (e.g., icon-based pairs vs. text-based pairs).

## 3. Results

### 3.1. Sample

**Figure 3:**
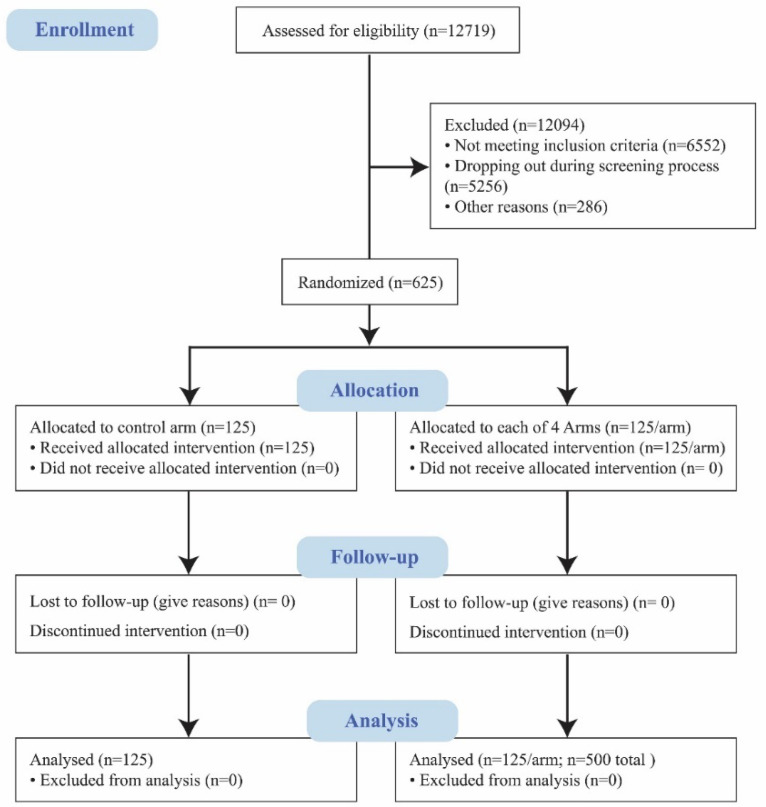
CONSORT Flow Diagram.

A total of 12,719 participants were contacted and screened for the eligibility criteria. Of these, 12,094 were excluded for not meeting the eligibility criteria (*n* = 6552), dropping out during the screening process (*n* = 5256), not meeting quality control (i.e., not being able to wear the eye-tracking glasses) (*n* = 271) or lack of consent (*n* = 15) (Figure 3).

A total of 625 participants of varying HL levels completed the study (Table 3).

### 3.2. Objective #1: Efficacy of FOPL Compared to Current Labeling

Participants were significantly more successful with FOPL than current labeling (control) for each task type and overall (Table 4). Participants in FOPL arms identified foods high in saturated fat, sugars, and/or sodium (findability tasks) significantly faster compared to control (current labeling).

Success rates and times to complete the shopping tasks varied as a function of HL (Table 5). Participants with adequate HL showed significantly greater successful decision-making for non-specific tasks. Participants at risk of marginal HL were significantly more successful in specific tasks with FOPL than with current labeling. Regardless of HL status, participants were significantly faster at successfully identifying foods high in nutrients of public health concern (findability task). Overall, for all five tasks, both marginal and adequate HL participants were significantly more successful with FOPL than with current labeling.

**Figure 4:**
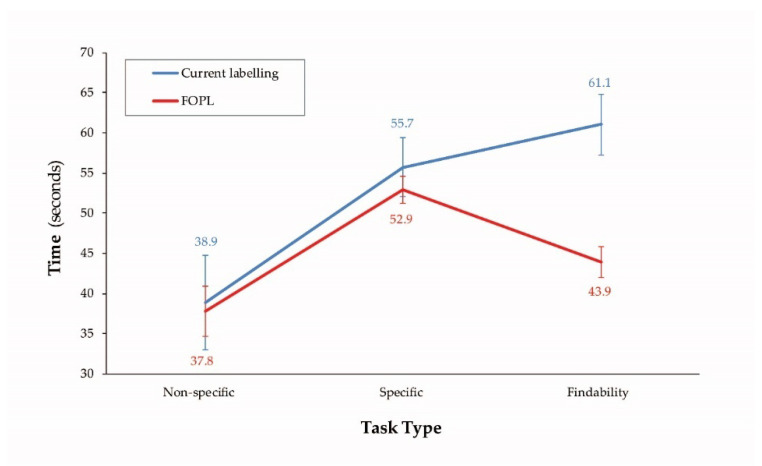
Successful decision-making time for current labeling vs. FOPL across task types.

Shopping success rates and decision-making times also varied with the complexity of the task (Figure 4). Participants took significantly more time (approximately 15 s) to make successful food choices to meet a specific dietary goal/need (specific task) than intrinsically motivated tasks (non-specific tasks) (*p* < 0.0001). Successful decision-making time for the control group increased with complexity of shopping tasks (i.e., specific and findability tasks). Time to successfully complete the non-specific and specific tasks was not statistically different between control and FOPL arms. However, those in the FOPL arms took significantly less time (approximately 17 s) to find foods high in saturated fat, sugars, and/or sodium (findability tasks) compared to control (current labeling).

### 3.3. Objective #2: Efficacy of Specific FOPL Designs

Shopping success rates and decision-making time were not significantly different between the four different FOPLs across all shopping tasks, for each task type, and for all 5 shopping tasks combined. Specific task outcomes showed significant differences, but further testing with Bonferroni’s adjustments did not identify pairs of FOPLs with statistically significant differences (Table 6).

### 3.4. Attentional Capture of FOPL Designs

For successful food decision making, for all five shopping tasks combined, there was no effect of FOPL design on time to first fixation (TTFF) (Table 7). The specific task outcomes showed significant differences, but further testing with Bonferroni’s adjustments did not identify pairs of FOPL with statistically significant differences.

**Figure 5:**
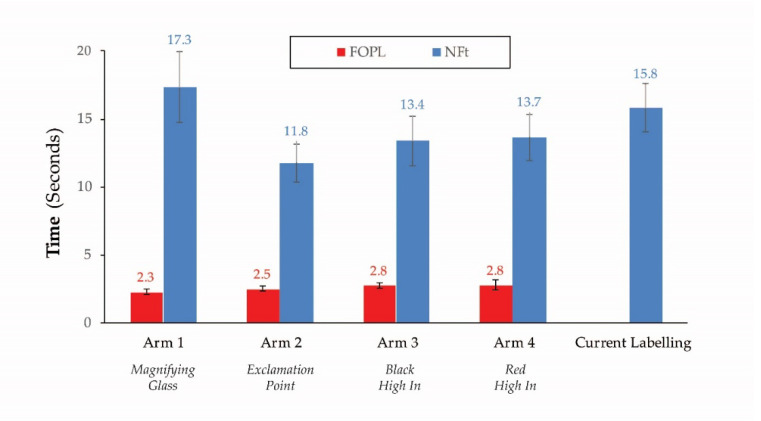
Visual processing and successful decision-making time.

Eye-tracking of time spent on FOPL when making successful food choices was not different between FOPL designs (*p*-value = 0.6) and was less than the time required to visually process the NFt across all four FOPL arms (Figure 5). While a significantly greater proportion of participants in the current labeling group compared to FOPL arms accessed the NFt (51% vs. 35%, Chi-squared *p*-value < 0.0001), time spent on the NFt was not significantly different between the four FOPL arms and current labeling (*p*-value = 0.3).

### 3.5. Food Decision-Making and Use of FOPL

Most participants stated that they used taste, brand name, and label information to make their food choices in each of the shopping tasks. In the absence of prior awareness of FOPLs on food packages, attentional capture and processing of the FOPL in the introductory soup-based task was reported by 24% of participants in the FOPL arms. Approximately one in five of these participants changed their soup selection after becoming aware of the FOPL (Arm 1: 20%; Arm 2: 23%; Arm 3: 22%; Arm 4: 20%; no significant differences between the arms). Regardless of the labeling approach, those who were successful in their food choice tasks relied more on nutrition information while those who failed tended to rely more on taste (Table 8). Self-reported usage of nutrition information for successful decision making was significantly greater in FOPL arms than with current labeling (*p* < 0.0001).

Overall, the FOPLs were used to compare foods within the same category, to choose foods that did not carry a FOPL, and to select healthier options for non-specific tasks and specific tasks. Participants relied on the FOPL to identify foods high in saturated fat, sugars, and/or sodium (Table 9).

**Figure 6:**
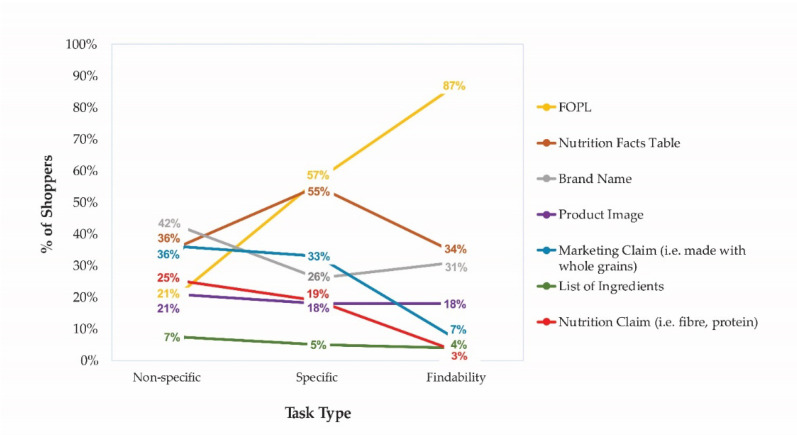
Reliance on FOPL in successful decision making (all FOPLs).

Among all FOPL arms, self-reported consumer reliance on the FOPL to make successful food choices increased as the cognitive burden of the tasks increased (i.e., specific and findability tasks) while reliance on nutrition and marketing claims decreased. Industry labeling (brand name and marketing claims) was relied on more frequently when performing non-specific food tasks (Figure 6).

## 4. Discussion

Recent Canadian research supports the development and refinement of nutrition labelling to meet the needs of all Canadians, and more importantly, those facing constraints due to limited/marginal health literacy [5]. This study, using a validated health literacy assessment tool recently adapted for use in Canada [40], provides empirical support for the effectiveness of “high in” nutrient specific FOPL to help Canadians of varying HL levels make healthier food choices and more easily identify foods high in sugars, sodium, and saturated fats. These findings add to recent Canadian research testing the efficacy of a nutrient specific “High in” FOPL, to steer consumers to healthier beverage and snack purchases [41], by also addressing the efficacy in commonly consumed foods used as part of a meal (e.g., cereal, soup, yogurt). This is the first study to objectively assess and demonstrate the efficacy of “high in” nutrient specific FOPL on pre-packaged foods as a quick and easy labeling tool for people of varying HL levels to make healthier and more informed choices with respect to sugars, sodium, and saturated fats. This Canadian study adds to the growing global body of research on the efficacy of a “high in” nutrient specific FOPL approach [28,42,43,44,45,46,47,48,49,50,51] and confirms its potential as a labeling approach to discourage the purchase and consumption of foods high in nutrients of public health concern.

Current food label information creates many challenges for food-based decision-making [5,6]. To be effective as a population health tool, a FOPL must enable people of varying HL levels to make healthier food choices and identify foods high in nutrients of public health concern. From a policy and regulatory perspective, an effective FOPL should give quick and easy guidance to accomplish these tasks. These objectives were accomplished by reducing the cognitive load for food-based decision-making, in particular for those Canadians disadvantaged by risks of marginal or limited HL, who struggle with the Nutrition Facts table (NFt), a key labelling component on pre-packaged foods [5,6,7]. Addition of the nutrient specific “High In” FOPL effectively reduced the cognitive burden and facilitated healthier food choices among those at risk of marginal HL. The cognitive burden of the FOPL was significantly smaller than the NFt, as evidenced by the lower amount of decision-making time spent on the FOPL. Not surprisingly, the nutrient specific “high in” FOPLs effectively discouraged participants of varying HL levels from choosing foods high in nutrients of public health concern and helped them to successfully identify foods high in sugars, sodium, and/or saturated fat more quickly than with the current NFt labeling.

An effective FOPL requires attention to and comprehension of the information being conveyed in the varied contexts in which people engage with it. In the absence of previous exposure to the FOPL, we questioned whether or not participants might verify their understanding of the FOPL by accessing familiar, credible nutrition information (i.e., NFt) even though the cognitive burden would be greater. The eye-tracking data showed that fewer participants referred to the NFt in the FOPL arms, suggesting a certain level of confidence in their ability to make successful decisions with less reliance on the NFt. In structured interviews exploring consumer understanding of label information, the presence of the FOPL was influential in driving a greater overall use of nutrition information when making successful food choices as compared to current labeling. Participants motivated to specifically lower their intakes of sugars, sodium, and/or saturated fats took significantly more time to successfully select foods than when choosing foods to meet non-specific, intrinsic motivations. While the FOPL may have helped participants to decrease the choice set of foods under consideration for specific tasks, their use of other nutrition information to evaluate their remaining choices effectively increased the cognitive burden and the time needed to process the additional information. In contrast, the findability tasks which required identification of foods high in nutrients of public health concern, were quickly accomplished with the addition of the “high in” FOPL on foods. Nonetheless, throughout the shopping tasks, participants’ self-reported reliance on the FOPL to make successful food choices increased as the cognitive burden of the tasks increased. While it is difficult to rule out whether the FOPL would have had even greater initial uptake and use if familiarity had been higher, these findings provide support for the “high in” FOPL approach as an accessible and easily understood tool for making healthier food choices.

FOPLs included in this study focused on particular design features: the value of color, as well as the presence of and nature (or tone) of pictograms. Eye-tracking data exploring attentional capture showed that all four FOPL designs efficiently captured consumers’ attention and were equally effective in helping consumers of varying HL levels evaluate food products to make successful food choices across all shopping tasks. In the absence of prior awareness and exposure to FOPLs on food packages, one in four participants among the FOPL arms noticed the FOPL when first exposed to it. However, with repeated exposure to FOPL, participants became more efficient at choosing healthier foods, lower in nutrients of public health concern, as well as identifying foods high in nutrients of public health concern, regardless of the FOPL design. Similar to other experimental studies with “high in” FOPL directives [28,47], these findings confirm that the presence of a “high in” FOPL can improve consumer understanding of excess sugar, sodium, and/or saturated fat content in food products from a variety of food categories and increase consumer capacity to identify those food products with excessive amounts of sugar, sodium, and/or saturated fat.

### Strengths and Limitations

Strengths of this study include the naturalistic setting in a retail food lab designed to replicate a typical grocery shopping experience and the integration of tasks for different motivational scenarios to test the efficacy of a “high in” FOPL to meet the varying dietary goals and needs of consumers, including making food choices based on personal motivations, choosing foods to meet pre-determined dietary needs, and identifying foods high in nutrients of public health concern. Exposure of participants in a FOPL arm to only one specific FOPL design throughout their shopping experience provided a more objective comparison by removing the bias of personal preferences. The use of a HL lens in recruitment and data analysis assessment tools ensures findings are applicable to those Canadians disadvantaged by marginal HL. This study highlights the greater challenge they faced when relying on current labelling to make food choices compared to those with adequate HL.

Some limitations of this study need to be mentioned. The sample size of the study was established to answer the primary objectives. Multiple testing for secondary objectives must be looked at with caution and should be used for hypotheses generation for future studies. Pre-implementation studies such as this one have relied on a brief exposure to the FOPL, yet in a real world setting consumers would be frequently exposed to the FOPL when interacting with food packages both in the retail environment and at home. Although this study was conducted in a naturalistic supermarket setting, there was no exchange of money or food products and participants were aware that they were participating in a research study, which may have led to a change in food-related behaviors. It is also possible that wearing eye-tracking glasses and being asked questions about the food labels may have influenced participants to pay more attention to the food labeling when making their food choices.

While this research study demonstrates the efficacy of a nutrient-specific warning label on foods for assisting shoppers in recognizing healthier foods as well as what not to buy, there is no hard evidence that this will translate into an actual impact on their shopping behavior. A post-implementation evaluation of mandatory FOPL approaches supported by awareness and educational campaigns and frequent exposure would generate an assessment of effectiveness in real-world conditions, however, these evaluations would be limited by the lack of a comparator group (i.e., control or other FOPL designs). Future studies integrating global comparisons of mandatory FOPL approaches would help nutrition policy makers better understand how different FOPL approaches may be able to achieve healthy eating objectives.

## 5. Conclusions

These research findings add to the growing body of research on the efficacy of a “high in” nutrient-specific FOPL approach as a useful tool for enhancing the capacity of consumers of varying HL levels to make healthier food choices and identify foods high in nutrients of public health concern [28,42,43,44,45,46,47]. Of particular importance is the value of this “high in” FOPL approach for those consumers limited by risks of marginal HL who face challenges using nutrition information to make food choices.

## Figures and Tables

**Table 1:** Shopping task descriptions.

Type of Shopping Tasks	Shopping Task Objectives	Details of Shopping Task	Task Example
Introductory task (1 task—soup)	To introduce participants to the eye-tracking technology. It also introduced participants in the FOPL arms to the FOPL.	In this introductory task, ALL participants were asked to shop for a soup for their household.	Choose a soup for your household.
Non-specific shopping task (1 task)	(1) To determine if FOPL on pre-packaged foods are more effective than current labeling when relying on consumers’ intrinsic motivation for making healthier food choices. (2) To compare efficacy between FOPL arms.	Participants shopped in an assigned food category and chose a food for their household.	Choose a yoghurt for your household.
Specific shopping task (2 tasks)	(1) To determine if FOPL on pre-packaged foods are more effective than current labeling when choosing foods to reduce intakes of saturated fat, sugars, and/or sodium. (2) To compare efficacy between FOPL arms.	Participants shopped in an assigned food category and chose a food for their household to reduce intakes of saturated fat, sugars, and/or sodium.	Choose cereal for someone trying to cut down on their sugar intake.
Findability task (2 tasks)	(1) To determine if FOPL on pre-packaged foods are more effective than current labeling at helping consumers identify foods high in saturated fat, sugars, and/or sodium. (2) To compare efficacy between FOPL arms.	Participants shopped in an assigned food category to find a food high in saturated fat, sugars, and/or sodium.	Find any cracker high in sodium and saturated fat.

**Table 2:** Shopping task layout.

Food Categories	TASK TYPE		
	Selection of Food without Replacement from Task 1 Onwards		
	Tasks 1 and 2	Tasks 3 and 4	Tasks 5 and 6
	2 Non-Specific Task	2 Specific Task	2 Findability Task
	Options	Options	Options
	Task 1		
	Introductory		
Soup	Soup		
	Task 2		
	Non-Specific Task		
Cereal	Cereal	Low sugar	High sugar
Yogurt	Yogurt	Low sugar	High satfat
		Low (sugar + satfat)	High (sugar + satfat)
Crackers OR	Cracker	Cracker	Cracker
		Low sodium/Low satfat	
Crackers with Cheese		Cracker with Cheese	High sodium/High satfat
		Low satfat	
Salad Dressing	Salad dressing	Low sodium	High satfat
		Low (sodium + satfat)	High (sodium + satfat)
Beverage	Beverage	Low sugar	High sugar
Bar			High sugar
			High satfat
			High (sugar + satfat)

**Table 3:** Socio-demographic characteristics of participants.

		Control Current Labeling	Arm 1 Magnifying Glass	Arm 2 Exclamation Point	Arm 3 Black High In	Arm 4 Red High In
	# of Participants:	*n* = 125	*n* = 125	*n* = 125	*n* = 125	*n* = 125
Gender	Male	62	62	58	59	62
	Female	63	63	67	66	63
Age	16 to 18	5	4	4	4	3
	19 to 24	6	13	9	12	11
	25 to 34	30	26	25	29	27
	35 to 49	38	33	33	31	30
	50 to 64	30	34	33	32	34
	65+	16	15	21	17	20
Populations of Interest	General Population	104	108	105	103	104
	Indigenous	7	6	6	6	6
	Teens	4	4	4	4	3
	Francophone	10	7	10	12	12
Health Literacy	Adequate	49	49	50	50	50
	Limited/Marginal	76	76	75	75	75

**Table 4:** Success rates (correct choices) and time to complete shopping tasks: control (current labeling) vs. front-of-package label (FOPL).

Shopping Task	Measure	Control *N* = 125	FOPL *N* = 500	* *p*-Value
Non-specific (1 task)	Success rate, *n* (%)	36 (28.8)	218 (43.6)	0.0029
	Time, seconds; mean ± SEM	28.5 ± 2.4	31.7 ± 1.7	0.92
	Success only	38.9 ± 5.9	37.8 ± 3.2	0.28
Specific (2 tasks)	Success rate, *n* (%)	184 (73.6)	858 (85.8)	<0.001
	Time, seconds; mean ± SEM	54.3 ± 3.3	52.3 ± 1.6	0.54
	Success only	55.7 ± 3.7	52.9 ± 1.7	0.36
Findability (2 tasks)	Success rate, *n* (%)	168 (67.2)	883 (88.3)	<0.001
	Time, seconds; mean ± SEM	64.7 ± 3.9	46.6 ± 1.9	<0.0001
	Success only	61.1 ± 3.8	43.9 ± 1.9	< 0.001
All 5 tasks	Success rate, *n* (%)	388 (62.08)	1959 (78.36)	<0.001
	Time, seconds; mean ± SEM	53.3 ± 2.9	46.1 ± 1.5	0.29
	Success only	56.4 ± 3.3	47.1 ± 1.6	0.02

* *p*-values from repeated measures statistical modeling, adjusting for HL, task type, and order for all 5 tasks; *n* = number.

**Table 5:** Success rates (correct choices) and time to successfully complete shopping tasks: by HL status.

		Marginal HL *N* = 377			Adequate HL *N* = 248		
Shopping Task	Measure	Control *N* = 76	FOPL *N* = 301	* *p*-Value	Control *N* = 49	FOPL *N* = 199	* *p*-Value
Non-specific (1 task)	Success rate, *n* (%)	24 (31.58%)	128 (42.52%)	0.08	12 (24.49%)	90 (45.23%)	0.008
	Time (sec) mean ± SEM	39.1 ± 8	38 ± 4.7	0.30	38.3 ± 8	37.4 ± 3.7	0.48
Specific (2 tasks)	Success rate, *n* (%)	106 (69.74%)	512 (85.05%)	<0.001	78 (79.59%)	346 (86.93%)	0.065
	Time (sec) mean ± SEM	58.5 ± 5.6	52.7 ± 2.3	0.33	51.9 ± 4.2	53.2 ± 2.3	0.87
Findability (2 tasks)	Success rate, *n* (%)	96 (63.16%)	522 (86.71%)	<0.001	72 (73.47%)	361 (90.70%)	<0.001
	Time (sec) mean ± SEM	62.8 ± 5	43.8 ± 2.6	<0.001	58.7 ± 5.8	43.9 ± 2.8	0.002
All 5 tasks	Success rate, *n* (%)	226 (59.47%)	1162 (77.21%)	<0.001	162 (66.12%)	797 (80.1%)	<0.001
	Time (sec) mean ± SEM	58.3 ± 4	47.1 ± 2	0.06	53.9 ± 3.7	47.2 ± 2.1	0.31

* *p*-values from repeated measures statistical modeling, adjusting for HL; task type and task order for all five tasks.

**Table 6:** Success rates and time to complete shopping tasks: specific FOPL designs.

Shopping Task	Measure	FOPL Designs				
		Arm 1 Magnifying Glass	Arm 2 Exclamation Point	Arm 3 Black High In	Arm 4 Red High In	* *p*-Value
Non-specific (1 task)	Success rate *n* (%)	52 (41.6%)	55 (44.0%)	53 (42.4%)	58 (46.4%)	0.88
	Time (sec) mean ± SEM	26.3 ± 2.2	34.9 ± 4.4	32.8 ± 3.2	33.1 ± 3.1	
	Success only	27.9 ± 3.2	43.5 ± 9.1	43.2 ± 6.2	36.3 ± 5.2	0.27
Specific (2 tasks)	Success rate *n* (%)	211 (84.4%)	216 (86.4%)	214 (85.6%)	217 (86.8%)	0.88
	Time (sec) mean ± SEM	51.8 ± 3.2	55.1 ± 3.4	50.9 ± 3.2	53.4 ± 3.4	
	Success only	51.9 ± 3.5	56.2 ± 3.6	51.2 ± 3.2	52.1 ± 3.2	0.66
Findability (2 tasks)	Success rate *n* (%)	225 (90.0%)	219 (87.6%)	225 (90.0%)	214 (85.6%)	0.40
	Time (sec) mean ± SEM)	44.1 ± 3.3	50.9 ± 4.9	47.1 ± 3.9	44.4 ± 3.4	
	Success only	43.4 ± 3.2	44.9 ± 4.5	44.5 ± 3.8	42.6 ± 3.8	0.90
All 5 tasks	Success rate *n* (%)	488 (78.08%)	490 (78.40%)	492 (78.72%)	489 (78.24%)	0.99
	Time (sec) mean ± SEM	43.6 ± 2.6	49.4 ± 3.6	45.7 ± 3.1	45.7 ± 2.9	
	Success only	45.4 ± 2.8	49.7 ± 3.5	47.3 ± 3.2	46.1 ± 3.1	0.78

* *p*-values from repeated measures statistical modeling, adjusting for HL (and task type and task order for all five tasks).

**Table 7:** Time (seconds) to first fixation on FOPLs for varying shopping tasks.

Shopping Task	Time to First Fixation TTFF (Seconds)	FOPL Designs				
		Arm 1 Magnifying Glass	Arm 2 Exclamation Point	Arm 3 Black High In	Arm 4 Red High In	* *p*-Value
Non-specific (1 task)	TTFF success + fail	8.12 ± 1.1	6.49 ± 1.0	8.43 ± 1.2	9.18 ± 1.1	0.14
	TTFF success only	8.21 ± 1.8	7.01 ± 2.2	5.66 ± 0.9	8.7 ± 1.8	0.35
Specific (2 tasks)	TTFF success + fail	11.35 ± 1.2	9.08 ± 0.9	12.76 ± 1.3	9.12 ± 1.1	0.03
	TTFF success only	11.65 ± 1.3	8.98 ± 0.9	12.6 ± 1.3	9.2 ± 1.2	0.03
Findability (2 tasks)	TTFF success + fail	10.52 ± 1.0	9.23 ± 1.1	10.74 ± 1.1	9.16 ± 1.3	0.39
	TTFF success only	9.18 ± 0.9	9.61 ± 1.2	10.12 ± 1.2	9.45 ± 1.4	0.83
All 5 tasks	TTFF success + fail	10.43 ± 0.7	8.66 ± 0.6	11.12 ± 0.9	9.15 ± 0.8	0.02
	TTFF success only	10.10 ± 0.8	9.08 ± 0.6	10.69 ± 1.0	9.22 ± 1.0	0.07

* *p*-values from repeated measure linear regression analysis (adjusting for task type and task order for all five tasks).

**Table 8:** Factors on which product choice is based—all shopping tasks combined (excluding intro task).

Product Factors	Control (Current Labeling) *N* = 125			All FOPL Arms *N* = 500		
	Success	Failure	*p*-Value	Success	Failure	* *p*-Value
Taste	44%	61%	<0.0001	41%	61%	<0.0001
Brand name	39%	55%	<0.0001	35%	48%	<0.0001
Perceived healthiness	31%	25%	0.02	23%	22%	0.46
Nutrition information	48%	28%	<0.0001	66%	46%	<0.0001
Ingredients	22%	26%	0.06	14%	14%	0.97
Other	3%	2%	0.45	2%	2%	0.24

* *p*-values comparing success to failure groups from logistic regression analysis with repeated measures.

**Table 9:** Use of FOPL (all arms combined, *N* = 500) to make successful food choices.

Use of FOPL	Task Type			
	Non-Specific	Specific	Findability	* *p*-Value
Compare foods	70%	68%	78%	<0.001
Choose foods without FOPL	69%	68%	N/A	0.9
Choose healthier foods	40%	30%	N/A	0.07

* *p*-values from logistic regression analysis with repeated measures.

## Supplementary Materials

The following are available online at https://www.mdpi.com/2072-6643/12/10/3199/s1, Figure S1: Example of current labelling vs customized elements in design mock ups for FOP symbol arms.

## Abbreviations

AOI	Areas of Interest
FOPL	Front of Package Label
HL	Health Literacy
GTA	Greater Toronto Area
HES	Healthy Eating Strategy
LOI	List of Ingredients
NFT	Nutrition Facts Table
TCPS	Tri-Council Policy Statement
TTTFF	Time to First Fixation
WHO	World Health Organization
