# Supplementary material for: Efficacy of “High in” Nutrient Specific Front of Package Labels—A Retail Experiment with Canadians of Varying Health Literacy Levels
Source: Nutrients. 2020 Oct 20;12(10):3199. doi: 10.3390/nu12103199 (PMC7590840; doi:10.3390/nu12103199)
Supplement: Supplementary file 1 [file nutrients-12-03199-s001.pdf]

Food Package Stimuli

Seven food categories (cereal, fruit beverage, yoghurt, bars, soup, salad dressings, and crackers) were chosen to represent a wide range of portion sizes and food uses (i.e., part of a meal or snack, treat, condiment). Each of the base foods required regulated labelling information and included FOP labels, where appropriate, based on Health Canada's proposal. Health Canada provided a list of products to include for each of the seven food categories in the research. Each product listed included detailed specifications on the type of FOP symbol that should be used, including size level of the FOP symbol (1-6), dimensions of the FOP symbol, and the 'High In' nutrients within the FOP symbol. Additionally, on a product by product basis, changes were applied to the existing package design to align with FOP symbol guidelines. An example of this type of individualized package customization would be to ensure that existing health or nutrient content claims on pack did not supersede the claims made in the FOP symbol through relocation on front of package and/or reduction in size (see Figure 1. below).

Figure 1. Example of current labelling vs customized elements in design mock ups for FOP symbol arms

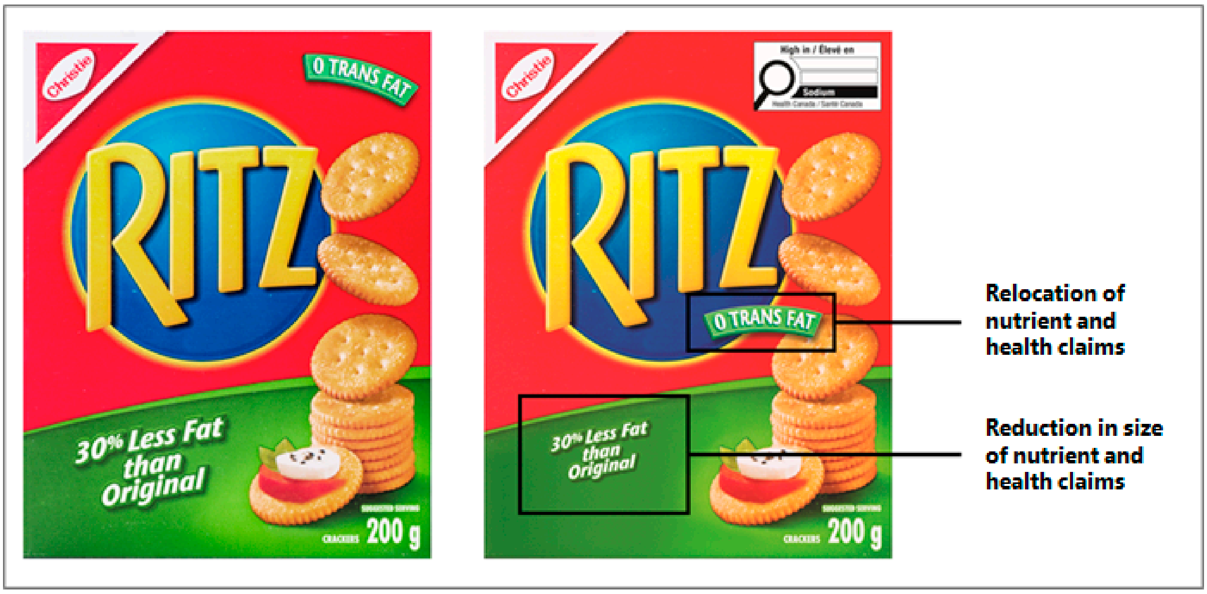

The FOP symbol and additional customizations were applied across all applicable packages in the category and jpeg proofs were provided to Health Canada for approval. Once approvals were received, applicable substrate was selected (i.e., glossy or matte) and an opaque label was produced. Each label was custom cut to fit to the relevant panel(s) of the product. In most cases, the new label replaced or covered the existing front panel of the package. Where bilingual packaging had 2 sides (French and English), two labels were produced in the necessary language and affixed to the product. In cases where a continuous label was present, e.g., soup, a continuous new label was produced and affixed to the existing product.
